# Examining the U-shaped relationship between non-agricultural sources water pollution and the urban-rural income gap

**DOI:** 10.1371/journal.pone.0305530

**Published:** 2024-07-18

**Authors:** Dong He, Zhongyuan Sheng, Chunxiao Tian

**Affiliations:** 1 School of Management, Xihua University, Chengdu, Sichuan, China; 2 School of Economics, Xihua University, Chengdu, Sichuan, China; Khwaja Fareed University of Engineering & Information Technology, PAKISTAN

## Abstract

Determining how the economy and society interact with the environment of water quality is essential to determining the financial impact of green development. Based on China’s provincial panel data from 2010 to 2021, this research considers non-agricultural sources of water pollution (NASWP) as a negative factor of production, investigates its influence on the urban-rural divide, and explains the mechanism of action. The empirical results show that there is a significant correlation between NASWP and the urban-rural gap, with a "U-shaped" relationship between the two. Water pollution first reduces and then increases the urban-rural income gap, and the results are robust after considering endogeneity. Mechanistic research demonstrates that NASWP cause a loss in food output, which in combination with changes in food prices and food subsidy programs impacts the incomes of rural dwellers, thereby having an influence on the urban-rural income gap. Using the threshold effect model, it is discovered that under the combined influence of agricultural mechanization and food subsidy policy, the relationship between NASWP and urban-rural income divide exhibits an U-shape in areas with high agricultural mechanization and an "inverted U" shape in areas with low agricultural mechanization.

## Introduction

Water is not only necessary for biological living, but it is also extensively and intensively used as a production element. In desert and water-scarce places, plentiful and clean water supplies are extremely important. Along with increasing economic and social growth, water contamination has become a major source of worry for society as a whole. More than 30% of the world’s population lives without any sort of sanitation, and 90% of sewage in underdeveloped nations is released into water bodies without being treated [1]. However, pollution is not limited to agricultural sources; effluent discharges from industry and home usage can also result in negative environmental externalities. According to 2024 United Nations water resources assessment report, pollution discharged upstream from industrial production centers can reduce economic growth by 30 percent, and the information technology industry, which manufactures semiconductors, circuit boards, and batteries, also releases metal pollutants in wastewater or through e-waste leaching, which can have long-term effects on rivers and lakes, disrupting ecosystems and jeopardizing human health. The scarcity of clean water resources has indisputably exerted a global impact [2].

The theory of externality posits that the discharge behavior resulting from production and business activities of enterprises possesses strong negative externalities, adversely affecting the ecological environment and leading to the loss of social welfare [3]. In regions where water scarcity for agricultural production is acute, groundwater and reclaimed water are commonly utilized as irrigation sources to alleviate water stress. However, this practice heightens the risk to freshwater resources essential for agricultural production [4].

The process of production and quotidian activities invariably yields wastewater of three distinct types: industrial, agricultural, and domestic, each carrying its unique set of challenges. This wastewater, particularly agricultural effluents, is the inadvertent consequence of agricultural enterprises, typified by utilization of fertilizers and pesticides, as well as dispersion from livestock and poultry farming. These effluents pose significant threats to both water quality and soil fertility. In the context of China’s water-management landscape in 2022, it is noteworthy that agricultural water consumption outpaces industrial water consumption by a factor of approximately 3.9. However, paradoxically, the value addition from the primary or agriculture-based sector constitutes a mere 18.3% of the secondary or manufacturing sector. A closer analysis reveals that a preponderance of industrial activities and domestic routines do not contributory favorably to the augmentation of agricultural output value. Simultaneously, the wastewater discharged from these activities is imbued with a panoply of pollutants encompassing Chemical Oxygen Demand (COD), total nitrogen, total phosphorus, ammonia nitrogen, and heavy metals. And, they were overinvested in groundwater [5].

Segments of NASW find their way into rural sectors via hydrologic circulation corridors, including but not limited to rivers, lakes, and subterranean aquifers, thereby establishing a conduit for pollutant migration from urban to rural arenas via spatial translocation. The externality theory underscores that the behaviors exhibited by enterprises during their operational and manufacturing activities espouse significant negative externalities. These detrimental externalities, in turn, deliver adverse repercussions to the ecological environment, generating social welfare detriments [3]. Investigations launched by the China Agricultural and Environmental Protection Research Institute during the middle of the 1980s reveal a notable decline in grain yield from farmlands irrigated with effluent, showcasing an average reduction of 210 kilograms per hectare compared to their counterparts irrigated with pristine water resources [6]. In countries and regions grappling with a formidable dearth of water for agricultural endeavors, alternative strategies, such as the use of groundwater and recycled water as irrigation sources, have emerged as prevalent solutions to counteract water scarcity [7]. However, this recourse improves the threat posed upon the freshwater components, which are indispensable to agricultural production.

Some scholars have described the current situation of water pollution from economic factors and discovered that various factors such as the industrialization rate, urban-rural income gap, agricultural production structure, financial support for agriculture, and natural conditions have differing efficiencies in agricultural three-dimensional pollution discharge across different regions [8, 9].Furthermore, agricultural pollution loads tend to grow faster in low-income countries [10]. Regarding the impact of income disparity on water pollution, some scholars argue that its effect on environmental quality varies across countries with different income levels. When focusing on water pollution specifically, scholars have observed that the abundance of water resources moderates the relationship between income and water pollution [11]. Additionally, pollution exacerbates health and livelihood risks for disadvantaged groups and widens inequality in countries with higher-than-average GDP per capita or lower-than-average natural or human capital [12].

The established literature provides a richer discussion of the relationship between income disparity and water pollution. Existing studies have concluded that environmental pollution has both psychological and physiological effects [13]. This pollution reduces labor productivity [3] and labor supply [14]. In cases of regional differences in labor supply, environmental pollution serves as the primary pathway for transmitting the income gap [15]. Consequently, a transmission pathway of "pollution-health-labor-income gap" has been established [16–19].

However, certain limitations persist in the current body of research. Existing studies primarily concentrate on air pollution when assessing environmental pollution, whereas research from the perspective of water pollution remains relatively sparse. Water resources, as a crucial necessity for human survival, play a pivotal role in both agricultural food security and industrial economic development. The increase in water pollution has also imparted significant negative externalities on the economy and society. Despite its profound impact, the effect of water pollution on the economy and society has garnered less attention compared to air pollution, which is more easily observable and perceptible.

From an expenditure perspective, the costs of diet and health increase equivalently for both urban and rural residents. Regardless of their geographical location, the agricultural products consumed by these residents are primarily sourced from the agricultural sector. Assuming comparable product quality, a reduction in NASWP would lead to an increase in supply, potentially resulting in both lower prices and improved quality. This phenomenon would affect the cost of diet and health uniformly across all individuals. Turning to the income dimension, agricultural production holds greater significance but lesser stability in rural areas compared to urban areas [20]. Consequently, the impact of water pollution on the income of rural residents is more significant than that on urban residents.

In the initial stage of pollution, the supply of agricultural products may decrease, which may lead to a slight increase in prices. However, an escalation in prices may not necessarily translate into a pronounced drop in the demand for agricultural products among residents. This phenomenon can be attributed not only to the indispensable nature of agricultural products for survival but also to two other factors contributing to the persistence of this situation. Firstly, demand remains relatively stable as residents’ living needs and consumption patterns have not undergone notable changes due to water pollution [21]. Secondly, high-income individuals tend to be less price-sensitive, and given that the full impact of water pollution on agricultural product quality has not yet materialized, urban consumers may continue to purchase these products at their original prices, thus maintaining market demand [22].

**Fig 1:**
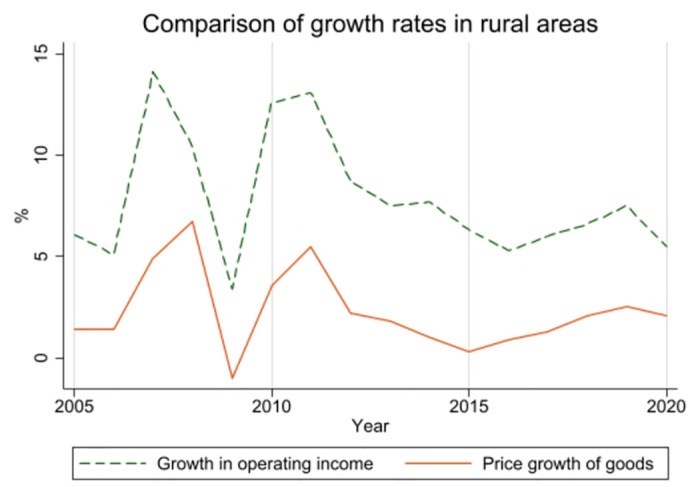
Comparison of farm household incomes and price volatility of agricultural products.

The operating income of rural residents constitutes a significant portion of their total income. This income primarily derives from agricultural activities, including the cultivation of crops such as grains, vegetables, and fruits, as well as the raising of livestock, poultry, and aquatic products. As illustrated in Fig 1, from 2005 to 2020, both the operating income of rural residents and the rural commodity price index in China demonstrated an upward trend with comparable fluctuations. Notably, the growth rate of rural residents’ operating income consistently outpaced that of the rural commodity price index. This trend indicates that the influence of rural commodity prices on farmers’ operating income has remained relatively stable over the observed period. These two data characteristics indicate that during the observation period, the impact of rural commodity prices on farmers’ operating income remains relatively stable.

In addition, non-agricultural source wastewater discharges create an externality problem. To address this, the government employs policy tools such as environmental taxes and ecological compensation. Based on the compilation of national agricultural product cost-benefit information by the National Development and Reform Commission of China, the average cash income from the cultivation of the three principal grains—rice, wheat, and corn—decreased to 481.7 yuan per mu in 2018 and then regained to 613.6 yuan per mu in 2020. According to the National Bureau of Statistics of China, the national average grain yield per mu in 2021 stood at 824 yuan, marking the highest level in nearly five years. From a cash income perspective, farmers derive their earnings from grain cultivation primarily through labor input and the utilization of their own land. Notably, according to the China Rural Revitalization Survey (CRRS) conducted by the Chinese Academy of Social Sciences, approximately 14% of the 304 administrative villages surveyed in 2021 solely viewed "rotation" as an ecological compensation policy. This policy provided a subsidy of up to 150 yuan per mu annually, accounting for roughly 18% of the total grain production revenue [23]. These measures aim to ensure that rural residents receive economic returns without undue risks [24], thereby helping to reduce the urban-rural income gap.

In the later stages of NASWP, the level of pollution in rural environments has been escalating due to the intensified cumulative effect of pollution, causing significant environmental damage [25]. Additionally, the discharge of wastewater from non-agricultural sources has continued to rise. In comparison to urban economic activities, which are less impacted by water pollution, this pollution not only significantly decreases crop yields and quality but also hikes farmers’ production costs, resulting in a decrease in their incomes. Cases of farmland losing its planting effectiveness due to factory wastewater discharge are not uncommon. Furthermore, addressing externalities through internalisation is often challenging, and farmers often do not receive adequate compensation for losses incurred due to pollution, ultimately leading to an unfair suppression of farmers’ incomes and an exacerbation of the urban-rural income gap.

This paper explores the influence of NASWP on the disparities between urban and rural areas in China, utilizing provincial panel data collated from 2010 through 2021. The robustness of this investigation’s empirical analysis hinges on a two-way fixed-effects model. Crucially, empirical evidence substantiates a remarkable non-linear correlation between NASWP and the gap in urban-rural incomes, exhibiting an undeniable U-shaped pattern. This signifies that the NASWP initially serves to narrow the income divergence, yet, subsequently, it contributes to a wider urban-rural income divide. The plausibility of endogeneity in our findings has been scrupulously accounted for and the resultant robustness further underscores the validity and solidity of this relationship. Our mechanistic hypothesis posits that NASWP trigger a downturn in grain yields, which in turn catalyzes the urban-rural income differential. Notably, a threshold effect model employed to evaluate heterogeneity indicates that the association between NASWP and the urban-rural income divide exhibits a differential pattern when mapped onto regions with disparate levels of agricultural mechanization. Specifically, areas replete with high levels of agricultural mechanization were found to conform to a U-shaped relationship, whereas an inverse U-shaped relationship was ascertained in regions disadvantaged with low agricultural mechanization levels.

The specific marginal contributions of this paper are threefold. Firstly, this article delves into the influence of water pollution originating from non-agricultural sources on the underlying transmission mechanism of the urban-rural income disparity, particularly through its impact on agricultural product sales revenue. This analysis is conducted from the perspective of environmental production factors. Furthermore, the rationale behind the emergence of the Environmental Kuznets Curve (EKC) is expanded to encompass non-agricultural sources, representing a fusion of EKC theory and externality theory. Secondly, prior studies have frequently observed diverse nonlinear relationships between environmental and economic variables, including U-shaped, N-shaped, and inverted U-shaped patterns. However, this article posits that the shape of the relationship curve between NASWP and the urban-rural income gap is contingent not solely on the variable itself but also on the context of varying production efficiencies. By integrating threshold effect models with the Logarithmic LMDI method, we demonstrate that this nonlinear relationship manifests distinct variations or even reversals under different levels of production efficiency. Thirdly, in terms of perspective, unlike prior studies that primarily focus on the consumer goods attributes of the environment, this paper considers water pollution as a negative production factor, elucidating its linkage with the urban-rural income gap from the supply-side perspective and thus shedding light on the causes of urban-rural income inequality from a production angle.

### Model construction and data description

#### Model construction

When utilizing panel data in a model, unobservable heterogeneity often exerts an influence on the explanatory variables. Two-way fixed-effects models effectively account for the interaction between the dependent variable and the explanatory variable across various time points, while also controlling for changes in the explanatory variable between different time periods. This approach helps to mitigate biases arising from omitted variables. Drawing upon the theoretical analysis and assumptions outlined in the preceding section, the STIRPAT model and LMDI method are both widely used econometric frameworks for empirical inquiries into environmental factors. In this paper, we build upon existing research [26] by incorporating the squared term of the explanatory variables to develop a hybrid LMDI-STIRPAT model, as presented in Eq (1).

$$Tl_{it}=\alpha_{0}+\alpha_{1}NASWP_{it}\text{+}\alpha_{2}{(NASWP_{it}\text{)}}^{\text{2}}+\alpha_{i}X_{it}+\mu_{i}+\gamma_{t}+\varepsilon_{it}$$ (1)

Where the explanatory variable *Tl*_*it*_ is the income gap between urban and rural residents in year *t* in region *i*; the core explanatory variable *NASWP*_*it*_ is the degree of non-agricultural sources of water pollution; *X*_*it*_ is a control variable indicating the economic characteristics of the region; *μ*_*i*_ and *γ*_*t*_ denote the region- and time-fixed effects, respectively; and *ε*_*it*_ is the error term.

In order to demonstrate that non-agricultural water quality pollution originating from surface sources impacts the urban-rural income gap through reductions in crop yield, this paper constructs Eq (2), employing per capita food production as a mediating variable to investigate the underlying mechanism.


$$Output_{it}=\alpha_{0}+\alpha_{1}NASWP_{it}+\alpha_{i}X_{it}+\mu_{i}+\gamma_{t}+\varepsilon_{it}$$ (2)

The threshold effect pertains to the occurrence of a significant change in a variable or system state when a particular level is attained by another variable. This transition is not gradual but abrupt, occurring precisely upon crossing a defined threshold. In the context of agricultural production, enhancing production efficiency involves advancements in technology, equipment, management, and various other domains. However, these advancements do not significantly influence resistance to water pollution until a certain threshold is met. Once production efficiency surpasses this threshold, agricultural production exhibits a marked increase in resilience against water pollution, attributed to more sustainable production methods, efficient resource utilization, and enhanced pollution management capabilities. This paper builds upon the research conducted [27] to validate the threshold effect associated with the total power of agricultural machinery through the establishment of a threshold effect model (3). This model systematically performs the sequential tasks of determining the threshold quantity, searching for the threshold value, and conducting a threshold effect regression.


$$\begin{array}{l} Tl_{it}=\alpha_{0}+\alpha 1NASWP_{it}*I(\text{AM}<{\text{th}}_{\text{1}})\text{+}\alpha_{2}{\left(NASWP_{it}\right)}^{\text{2}}*I(\text{AM}<{\text{th}}_{\text{1}})+\ldots \\ +\alpha_{n}*I(\text{AM}<{\text{th}}_{\text{n}})*NASWP_{it}+\alpha_{n+1}*I(\text{AM}<{\text{th}}_{\text{n}})*NASW{P_{it}}^{\text{2}}+\alpha_{i}X_{it}+\mu_{i}+\gamma_{t}+\varepsilon_{it} \end{array}$$ (3)

In the aforementioned equation, *I ()* is the indicator function, assuming a value of 1 when the condition within the parentheses is met and 0 otherwise. The variable *AM* represents the threshold variable, while *th* denotes the Nth threshold value.

*Selection of variables and data sources*. This paper examines NASWP and the urban-rural income gap across 30 provincial-level administrative regions in China from 2010 to 2022. Following the introduction of data sources and variable selection, descriptive statistics for the primary variables are presented. The raw data were primarily sourced from the China Rural Statistical Yearbook, China Environmental Yearbook, and China Statistical Yearbook. Missing values in the dataset were interpolated and filled in to ensure a balanced panel dataset. To mitigate the influence of scale, variables with excessively large values were normalized and log-transformed.

1. Explained variables. The selection of indicators for measuring the urban-rural income gap encompasses options such as the ratio of urban to rural disposable income per capita, the Gini coefficient, and the Theil index. The Theil index serves as a crucial analytical tool, widely utilized across economics, sociology, and policy analysis to assess and comprehend income inequality. Given that the Theil index demonstrates greater sensitivity to changes at both extremes of the income distribution and possesses robust characteristics, this paper adopts the methodology to construct the Theil index for measuring the urban-rural income gap [28], as outlined in Eq (4). In Eq (4), Z_it_ denotes the income of urban and rural residents, while Nit represents the urban and rural population. A higher Theil index indicates a wider urban-rural income gap, whereas a lower index signifies a narrower gap.
$$Tl_{\text{it}}=\sum_{i=1}^{2}\left(\frac{p_{ijt}}{p_{it}}\right)/\text{ln}(\frac{p_{ijt}}{p_{it}}/\frac{z_{ijt}}{z_{it}})$$ (4)
2. Explanatory variables. The principal explanatory variable is the degree of water pollution, represented as (*NASWP*). Chemical oxygen demand (*COD*) indicates the quantity of organic pollutants in the water, while total nitrogen and total phosphorus are primary contributors to eutrophication in aquatic environments. Ammonia nitrogen, furthermore, significantly influences the self-purification capacity and biological toxicity of water bodies [29]. An increase in the concentration of these pollutants corresponds to a more severe degree of water pollution. To capture the pollution status comprehensively and objectively, this study employs the entropy value method for a comprehensive assessment of these indicators. Drawing upon the methodology outlined, we analyze four common pollutants in wastewater: COD, total nitrogen, total phosphorus, and ammonia nitrogen. Data from each test sample collected during the calendar year are utilized to assess the severity of water pollution. Notably, all four pollutants serve as positive indicators in our analysis. Utilizing the entropy method, we determine the weights associated with each sample and calculate comprehensive scores, as previously described [30].
3. Channel variables. To assess the role of crop production as a conduit for water pollution to influence the urban-rural income gap, per capita food production (*Output*) was chosen as a mechanism variable for analysis. As a crucial indicator of agricultural production levels, per capita food production directly reflects the adverse effects of water pollution on agricultural production. Furthermore, agricultural production constitutes a significant source of income for rural residents, and any decline in food production directly impacts farmers’ income levels. Although urban residents are not directly involved in agricultural production, their food supply relies on agricultural output, and a decrease in food production can affect food prices, subsequently influencing the cost of living and food expenses for urban dwellers.
4. Threshold variable: The threshold variable is agricultural productivity (*AM*), which is measured by the natural logarithm of the total power of agricultural machinery. The total power of agricultural machinery has been found to significantly enhance food production [31]. Efficient agricultural production enables individual producers to till, irrigate, and harvest at a faster pace. Initially, the subsidy policy will be granted based on the actual planting area, irrespective of the grain output’s effectiveness. Farmers can earn subsidy income from this policy. However, in practice, there exists a mismatch between the subsidized area and the actual planting area [32]. Consequently, the buffering effect of the food subsidy policy in diverse regions may vary in terms of agricultural production efficiency.
5. Control variables: Agricultural factor inputs, pollution control, price fluctuations, and technological progress may affect both the urban-rural income gap and water pollution, and therefore, they should be controlled. Regarding agricultural factor inputs, pesticide use and agricultural water use are chosen as proxies. For pollution control investment, wastewater treatment investment and exhaust gas treatment investment serve as proxy variables. Price variables are represented by the industrial producer ex-factory price index and the agricultural product price index. Finally, the technological progress of a region is measured by the expenditures of large-scale industrial enterprises. The definitions of these variables are outlined in Table 1.

**Table 1:** Variable description.

Type	Variables	Code	Definition
**Dependent**	Urban-Rural Income Gap	Tl	Theil Index of urban-rural income in each province
**Explanatory**	Non-agricultural sources of water pollution level	NASWP	Composite score of four water pollutants constructed using the entropy method
**Mechanism**	Per Capita Grain Output	Output	Per capita grain output in each province for the current year
**Threshold**	Agricultural Production Efficiency	AM	Total power of agricultural machinery in each province for the current year
**Controls**	Pesticide Usage	PU	Pesticide usage in each province for the current year
	Agricultural Water Consumption	AWC	Agricultural water consumption in each province
	Wastewater Treatment Investment	IW	Investment in wastewater treatment in each province for the current year
	Exhaust Gas Treatment Investment	IG	Investment in exhaust gas treatment in each province for the current year
	Industrial Producer Ex-Factory Price Index	PPI	Relative measure of changes in the overall level of industrial product ex-factory prices
	Agricultural Product Price Index	API	Relative measure of changes in the price level of agricultural products sold by producers
	R&D Expenditure of Enterprises Above Designated Size	RD	R&D expenditure of enterprises above designated size in each province for the current year

### Empirical results and analysis

#### Descriptive statistics

The descriptive statistics of the primary variables are presented in Table 2. The minimum value of the interprovincial Theil index, which quantifies the urban-rural income gap, stands at 0.017 over the period from 2010 to 2022. Notably, the standard deviation has narrowed from over 0.1 to 0.04 when compared to studies with a broader temporal scope. Furthermore, the average degree of water pollution in China is 0.156, exhibiting significant regional disparities. By analyzing the comparison between the level of water pollution and the mean value of the Theil Index in 2015 and 2022, it becomes evident that there is a limiting trend in China’s non-agricultural source effluent discharge, suggesting a tendency towards the alleviation of the urban-rural income gap.

**Table 2:** Descriptive statistics.

Variables	N	Mean	S.D.	Min	Max
**Tl**	390	0.0870	0.040	0.017	0.21
**NASWP**	390	0.156	0.131	0.002	0.613
**Output**	390	479.5	422.6	13.35	2499
**AM**	390	3418	2924	93.97	13353
**PU**	390	1.572	0.787	0.0200	2.862
**AWC**	390	4.447	0.988	1.281	6.333
**IW**	390	9.589	1.510	2.773	12.60
**IG**	390	11.19	1.224	4.949	14.08
**PPI**	390	0.060	4.424	4.877	4.633
**API**	390	0.062	4.470	4.887	4.658
**RD**	390	14.35	1.381	10.53	17.28

**Fig 2:**
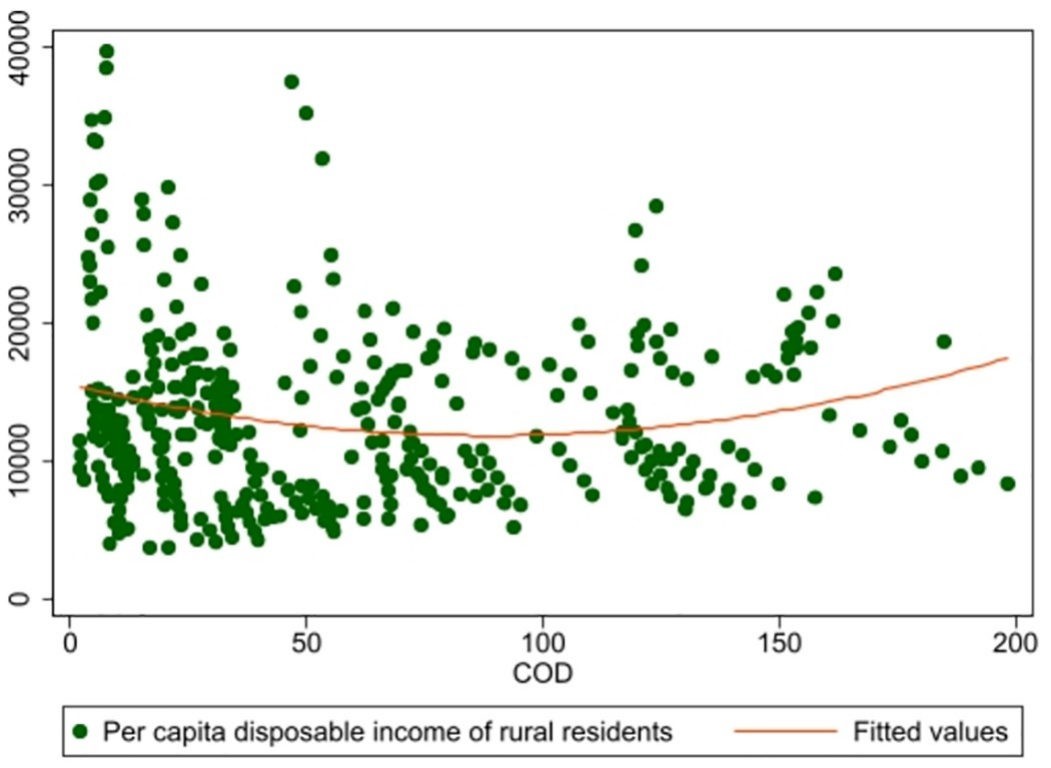
Non-linear relationship between NASWP and income of rural residents.

To provide a more intuitive understanding of the impact of pollutants in non-agricultural source wastewater on rural residents’ income, this paper presents a scatter plot in Fig 2, which demonstrates a U-shaped relationship between COD levels in NASWP and the urban-rural income gap.

#### Baseline regression results

Our investigation explores the hypothesized U-shaped association between NASWP and the divergence of earnings between urban and rural sectors. We employ a systematic, step-wise approach, drawing upon established research methodologies, to assess this relationship [33, 34]. In our first step, we conduct a basic linear regression analysis, juxtaposing the two key variables: NASWP and the urban-rural income disparity. Following this preliminary evaluation, we introduce an additional square term to our initial regression model, as delineated in Model (1). Through this quadratic regression analysis, we aim to discern the nuanced dynamics between these two interconnected facets. Subsequently, we scrutinize the results of our regression analysis, placing particular emphasis on the identification and assessment of the significance level of the quadratic coefficient within our regression model. If this entity is not only significant but also divergent from the generic quadratic coefficient, it suggests the presence of an U-shaped relationship nestled within our target variables. In the final phase of our investigation, we supplement and corroborate our quantitative analysis with visual data, embodied in the form of Fig 2. This provides a complementary perspective and a comprehensive picture of the complex interplay between NASWP and the urban-rural income gap, paving the way for nuanced interpretations and informed conclusions.

Column (1) in Table 3 manifests a positive correlation between the intensification of water contamination and the disparity in earnings between urban and rural settings. This correlation stems from a fundamental premise in environmental economics that ecological degradation invariably exerts a deleterious impact on economic proliferation, particularly in rural vicinities highly reliant on environmental resources for sustenance. Turning to the interpretation of Column (2), it encapsulates the findings while implementing the quadratic derivative of our explanatory metrics. The yielded results elucidate a negative association for the coefficient of water pollution and the Theil Index, while the coefficients for the quadratic term and Theil indicator manifest a positive relation. These coefficients, notably bearing inverse relations, attain statistical significance at the 10% threshold level. The narratives endure an evolution in Column (3), where a gamut of control elements has been incorporated. This strategic incorporation leaves undeterred the sign demarcations of the coefficient pertaining to the primary and secondary terms of the independent variables. However, it escalates the degree of significance, transcending from a modest 10% to a commendable 1%. Upon meticulously considering the derived findings, it is plausible to posit a "U" shaped relation trajectory. This deduction underpins the validation of an EKC existence in the peculiar dynamics of NASWP and the divergence of rural and urban income.

**Table 3:** Regressions of the urban-rural income gap on NASWP.

	Tl	Tl	Tl
	(1)	(2)	(3)
**NASWP**	0.0499***	-0.0553*	-0.0656***
	(6.2794)	(-1.9531)	(-2.7626)
**NASWP** ^**2**^		0.0693*	0.0923***
		(1.7383)	(2.8254)
**PU**			-0.0107
			(-1.5189)
**AWC**			-0.0230***
			(-3.7798)
**IW**			0.0007
			(0.8405)
**IG**			0.0011**
			(2.2080)
**PPI**			-0.0277***
			(-3.9427)
**API**			0.0149
			(1.4692)
**RD**			-0.0051
			(-1.3143)
**Province FE**	YES	YES	YES
**Year FE**	YES	YES	YES
**N**	360	360	360
**R** ^**2**^	0.8170	0.8431	0.8819

**Fig 3:**
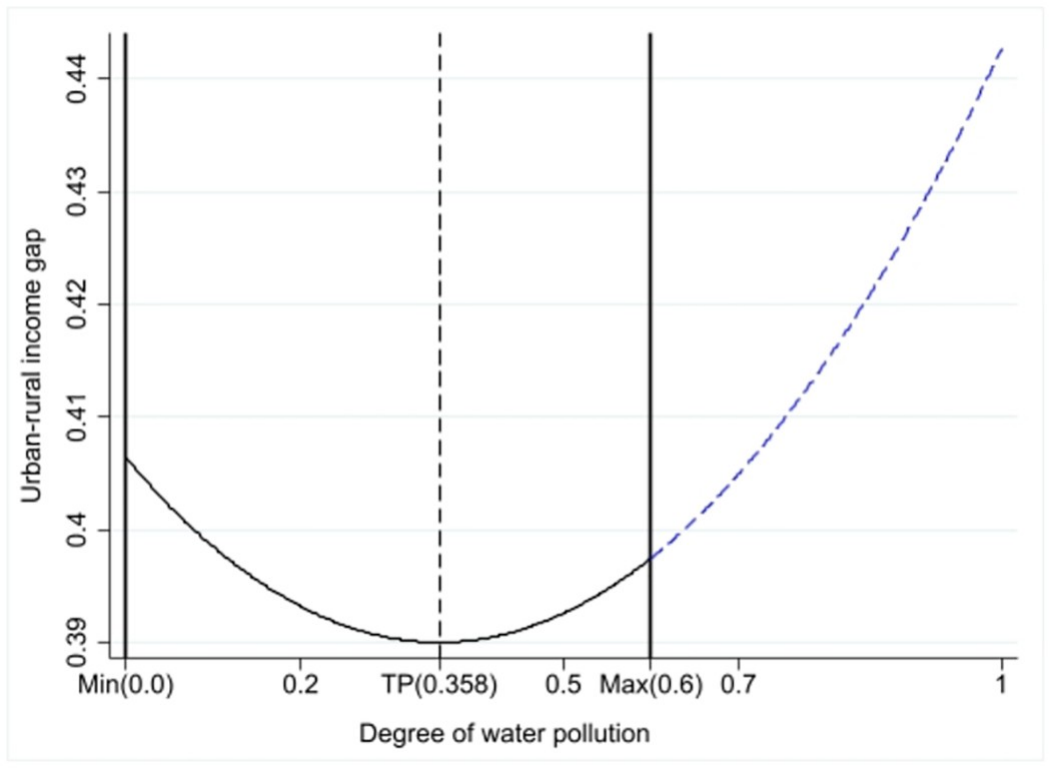
Graphical representation of the U-shaped relationship of the benchmark regression.

In an effort to fortify the persuasiveness of our conclusions, we have supplemented our paper with visual aids aimed at facilitating further validation of our findings. Included within these enhancements is a figure that illustrates the fitted curve delineating the relationship between NASWP and the urban-rural income disparity. The Fig 3. intuitively represents the complex interplay between these two variables, fostering a deeper understanding of our findings, and consequently enhancing the robustness of our research.

#### Robustness test

The omission bias arises from the exclusion of certain crucial variables from the model that have a bearing on both water pollution and the urban-rural income gap. Secondly, inaccuracies in measuring water pollution and the urban-rural income gap may arise due to various factors, including the type of monitoring equipment, the adopted methods, and the frequency of monitoring. Furthermore, water pollution can exacerbate the urban-rural income gap by compromising the health of the population and agricultural production, among other factors. Conversely, the widening of the urban-rural income gap can lead to unequal resource distribution and insufficient environmental protection investments, thus intensifying water pollution. This bidirectional causality is significant and cannot be overlooked. Therefore, this paper employs the instrumental variable method (2SLS) to address the potential endogeneity issues inherent in the model.

Before employing the instrumental variable method for endogeneity testing, this paper begins by drawing from the existing literature, adhering to the principles of selecting appropriate instrumental variables. The literature abounds with various instrumental variables used when pollution serves as a dependent variable. These include natural factors, such as the quantity of historical industrial effluent discharged in a region and the frequency of inverse temperature days [35]. Nevertheless, the selection of instrumental variables remains challenging in two respects.

Firstly, factors influencing the extent of water pollution are predominantly determined by the production sector, making it challenging to fully exogenously separate them from income inequality. Consequently, satisfying both the correlation with the independent variable and the exogeneity with the dependent variable simultaneously is problematic. Given this challenge, the approach adopted in this paper for constructing instrumental variables is based on natural geographical conditions, particularly the spatial distribution of rivers across cities [36]. This distribution can significantly impact the degree and speed of water pollution diffusion. Furthermore, forest coverage influences the greening of an area, carbon sequestration, and modifies the effectiveness of urban environments. Both these natural variables align with the hypothesis of a correlation with the explanatory variables. Additionally, while the natural geographical environment has a complex influence on the income of residents in a region, its impact on the urban-rural income gap is limited, thus adhering to the exogeneity hypothesis.

Secondly, the inclusion of a quadratic term in the model established in this paper poses the risk of encountering "regression exclusion zones" in the instrumental variables approach [37]. This arises because, in the second-stage regression, the linear projection of the quadratic term does not equate to the square of the linear projection of the primary term. Consequently, the quadratic term cannot be substituted with the square of the fitted value obtained in the first stage. Given this challenge, the approach adopted in this paper for constructing basic instrumental variables involves selecting several natural characteristic variables and creating interaction terms with the explanatory variables. Additionally, the explanatory variables and their quadratic terms are estimated simultaneously in a single stage. This refined approach ensures that the quadratic term is appropriately handled within the instrumental variables framework, enhancing the accuracy and reliability of the analysis.

Summary, this paper commences by calculating the national average of water pollution severity and its squared term. Subsequently, it calculates the pollution gap between observed provinces and the national average by subtracting the provincial pollution levels from the national mean. We then create the first instrumental variable (*IV1*) by multiplying the primary term with the river density of each province. The second instrumental variable (*IV2*) is derived by multiplying the secondary term with the forest area. The rationale for selecting *IV1* lies in the fact that river density serves as an indicator of water pollution dispersion. Although water resources volume is measured per capita and river density is shared, it does not directly impact the local urban-rural income gap, thus maintaining its exogeneity. The pollution gap in observed provinces complements the correlation, satisfying the instrumental variables assumptions. The choice of *IV2* is motivated by the squared term’s ability to mitigate the "regression exclusion zones" while forests, which aid in water pollution dilution, represent a public good. Similar to *IV1*, the exogeneity of *IV2* is justified. Consequently, *IV2* fulfills the requirements of an instrumental variable. In conclusion, both *IV1* and *IV2* are appropriately constructed to meet the criteria of instrumental variables, enabling accurate estimation and inference in our analysis.

The regression results are presented in Table 4. The initial two columns exhibit the ordinary least squares (OLS) estimations, both inclusive and exclusive of control variables. Notably, the correlation coefficients indicate a negative relationship between *NASWP* and *Tl*, whereas the correlation coefficients for *NASWP*^*2*^ and *Tl* are positive. These findings initially support the existence of a U-shaped relationship between NASWP and urban-rural income disparity. In contrast, Column (3) presents the second-stage regression outcomes from the two-stage least squares (2SLS) estimation. Here, the regression includes control variables and accounts for two-way fixed effects. Consistently, the correlation coefficients for *NASWP* and *NASWP*^*2*^ relative to Tl remain negative and positive, respectively. Both coefficients pass the 1% significance level test, further corroborating the existence of the aforementioned U-shaped relationship. In summary, the regression analysis provides robust evidence of a nonlinear association between water pollution and income inequality, with control variables and fixed effects taken into account.

**Table 4:** 2SLS regression results.

	Tl	Tl	Tl
	(1)	(2)	(3)
**NASWP**	-0.0840**	-0.0913***	-0.2398***
	(-2.2783)	(-3.0442)	(-3.3194)
**NASWP** ^**2**^	0.1046*	0.1276***	0.3816***
	(1.9625)	(3.0643)	(3.0694)
**Controls**	NO	YES	YES
**Province FE**	YES	YES	YES
**Year FE**	YES	YES	YES
**N**	390	390	390
**R** ^**2**^	0.8378	0.8787	0.8515

Table 5 presents the initial stage outcomes and various tests conducted for estimating the instrumental variable 2SLS. Both *IV1* and *IV2* exhibit significance with regards to the explanatory variables. *IV1* suggests that richer hydrological conditions can mitigate industrial effluent discharge through mechanisms such as recycling and adequate supply, while longer river lengths enhance the dispersal of water pollution. *IV2* indicates that extensive forested areas facilitate faster effluent purification. Consequently, it can be asserted that both *IV1* and *IV2* successfully meet the criteria of the first stage economic test.

**Table 5:** Test results for instrumental variables.

	(1)	(2)
	NASWP	NASWP^2^
IV1	-0.0002**	-0.0002*
	(-2.5419)	(-1.9535)
IV2	-0.0008***	-0.0004***
	(-5.5363)	(-3.8348)
Controls	Yes	Yes
Province FE	Yes	Yes
Year FE	Yes	Yes
LM statistic	17.171***	
F statistic	6.414	
	[4.58]	
Endogeneity test	7.405**	
N	390	390
R^2^	0.9150	0.8232

The regression results in column (3) of the second stage reveal no alteration in the U-shaped relationship between *NASWP*, *NASWP*^*2*^, and *Tl*. Furthermore, the endogeneity test achieves significance at the 5% level. The LM value rejects the null hypothesis of "insufficient identification of instrumental variables" at the 1% level. The F value (6.14) surpasses the critical value of the Stock-Yogo weak identification test at the 15% level (4.58), thereby rejecting the hypothesis of weak instrumental variables. These statistical tests have been successfully passed. Collectively, the findings indicate that the instrumental variables employed in this study are appropriately chosen, supporting the robustness of our estimations.

In this paper, we conduct a U-test on the aforementioned regression to ensure the robustness of the model’s regression results. Subsequently, we undertake a robustness test by employing two distinct methods: substituting the core explanatory variables, and replacing the explanatory variables.

The U-test results are shown in Table 6. The slope preceding the extreme value point is negative, whereas the slope following it is positive. Furthermore, the p-value is less than 0.01, indicating that the U-test has been successfully passed. This confirmation validates the robustness of the conclusion regarding the "U"-shaped relationship.

**Table 6:** U-test results.

	Lower bound	Upper bound	Overall test of presence of a U shape
**Extreme point**			0.276***
**Interval**	0.0017756	0.6133872	
**Slope**	-0.0908581	0.065166	
**t-value**	-3.043532	2.76088	2.59
**P>|t|**	0.0024658	0.0049461	0.00495

In addition to pollutants present in wastewater, the impact of air pollution on plant growth cannot be overlooked. This paper selects the natural logarithm of SO_2_ emissions and its square as the core explanatory variables for estimation. The estimation results presented in column (1) of Table 7 reveal that the coefficients of total SO_2_ emissions and its quadratic term, with regards to the urban-rural income gap, are significant at the 1% level. Notably, the signs of these coefficients are opposite to each other, indicating a "U-shaped" relationship between SO_2_ emissions and the urban-rural income gap. In the extant literature, the ratio of disposable income between urban and rural residents is commonly used as a metric for measuring the urban-rural income gap [38]. Given China’s specific context, urban residents typically enjoy significantly higher incomes than rural residents. A higher ratio thus signifies a wider urban-rural income gap. In this study, we adopt this ratio as an explanatory variable for the robustness test. The estimation results in column (2) of Table 7 demonstrate that, even when alternative measurement methods for the explanatory variable are employed, the degree of water pollution does not alter the U-shaped relationship between urban and rural income disparity.

**Table 7:** Robustness test results.

	Tl	Gap
	(1)	(2)
**NASWP**		-0.6106**
		(-2.5075)
**NASWP**_**2**_		0.8271**
		(2.4892)
**SO**_**2**_	-0.0129***	
	(-5.2813)	
**(SO**_**2**_ **)** ^**2**^	0.0014***	
	(3.5220)	
**Controls**	YES	
**Province FE**	YES	
**Year FE**	YES	
**N**	305	390
**R** ^**2**^	0.9201	0.8681

#### Analysis of transmission mechanisms

Pollutants present in wastewater, with their adverse implications, can trigger a considerable reduction in both the yield and quality of crops. Particular contaminants such as heavy metals are of significant concern due to their potential toxicity and elevated presence in both soil and crops. Such elements are capable of causing a notable decrease in photosynthetic capacity, which is a critical process for plant growth and development. Moreover, these detrimental pollutants typically find their way from the soil to the fruits and vegetables, following a transfer pathway that presents a direct threat to human health and environmental wellbeing [39, 40].

The detriments associated with agricultural surface water contamination predominantly stem from the excessive use of pesticides, fertilisers, and livestock excreta. These insidious pollutants infiltrate our surface water bodies through engagement in agricultural practices, resulting in a marked deterioration of water quality. Such degradation has profound implications, not merely distorting the optimal environment required for crop growth [41], but also instigating profound devastation within aquatic ecosystems. This, in turn, has cascading effects, ominously echoing throughout aspects of agricultural production [42]. Therefore, to ensure a robust agricultural yield, an unwavering supply of fertile soil and uncontaminated water resources is of paramount importance [43]. On the other hand, NASWP arises principally from the effluent discharges of both industrial entities and municipal wastewater treatment facilities. Such contaminants are exceedingly hazardous, encompassing heavy metals and a myriad of chemical substances. The water milieu, once contaminated due to improper waste disposal from factories and households, could invoke a series of plant afflictions [44]. These maladies inevitably translate into an alarming decrease in yield, posing a substantial threat to global food security. Thus, it becomes increasingly clear that managing surface water pollution, both agricultural and non-agricultural, is crucial to safeguarding our environment and sustaining agricultural productivity.

Mechanism tests are undertaken to investigate the potential channels through which NASWP impacts the urban-rural income gap. The regression results, presented in Table 8, reveal that NASWP significantly reduces food production at the 1% level, as demonstrated in column (2). When the per capita grain yield experiences a slight decline, farmers’ sales income derived from agricultural products increases owing to price hikes and the temporary stability of demand. Furthermore, the implementation of ecological compensation and agricultural subsidy policies has mitigated the urban-rural income gap. However, in scenarios where pollution levels become excessively severe, the sharp decline in agricultural product sales results in a significant reduction in sales income, which subsequently exacerbates the urban-rural income gap. The coexistence of these two scenarios establishes a non-linear relationship between NASWP and the urban-rural income gap, mediated by the linkage with crop yield.

**Table 8:** Mechanism test results.

	Tl	Output
	(1)	(2)
**NASWP**	-0.0656***	-0.6069***
	(-2.7626)	(-3.6972)
**NASWP** ^**2**^	0.0923***	
	(2.8254)	
**Controls**	YES	YES
**Province FE**	YES	YES
**Time FE**	YES	YES
**N**	360	360
**R** ^**2**^	0.8819	0.4585

#### Analysis of threshold effects

Table 9 shows the results of the determination of the number of thresholds, where the assumption of linearity is rejected at the level of 5% of the total power of agricultural machinery and the assumption of a single threshold cannot be rejected, so that the threshold variable can be considered to have a single threshold value.

**Table 9:** Number of thresholds established.

Variable	Threshold	RSS	MSE	F stat	Prob	Crit10	Crit5	Crit1
**AM**	Single	0.0120	0.0000	70.87**	0.0180	45.6830	55.3508	77.1635
	Double	0.0113	0.0000	22.74	0.4140	36.2197	42.3116	52.5718

Note: P-values and critical values were obtained from 500 simulations by Bootstrap method.

**Fig 4:**
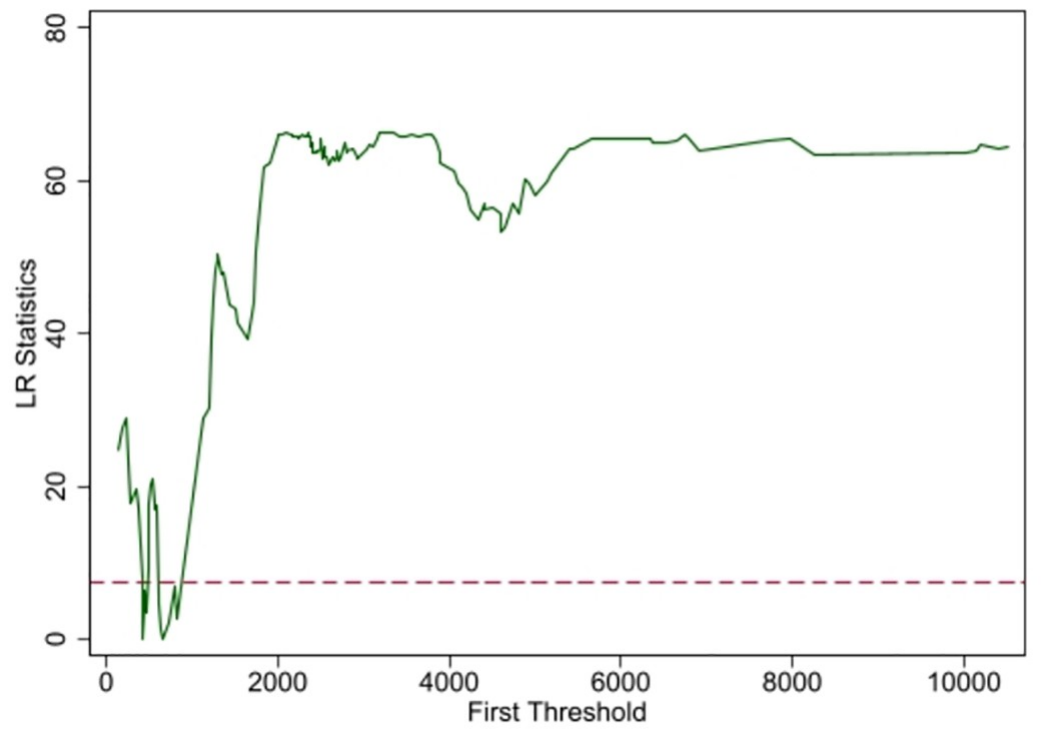
95% confidence interval for the threshold of total power of agricultural machinery.

After determining the number of thresholds to be 1, the specific values and their 95% confidence intervals are given in Table 10 and presented in Fig 4.

**Table 10:** Single threshold and its 95% confidence interval.

Model	Threshold	Lower	Upper
Th1	430.69	421.31	444.33

Table 11 presents the outcomes of the threshold effect regression. When agricultural mechanisation is at a low level, the coefficient of the explanatory variable stands at 0.6198, whereas the coefficient of its squared term is -11.4911. This suggests an "inverted U" shaped relationship between water pollution and the urban-rural income gap, contrary to the findings of the main effect regression.

**Table 11:** Threshold effect regression results.

	Tl
**NASWP (AM<th1)**	0.6311*
	(1.9221)
**NASWP2 (AM <th1)**	-12.3316***
	(-3.2951)
**NASWP (AM >th1)**	-0.0893***
	(-3.2101)
**NASWP2 (AM >th)**	0.1145***
	(3.2413)
**Th (95%Confidence interval)**	430.69
**Controls**	YES
**Province FE**	YES
**Year FE**	YES
**N**	390
**R** ^**2**^	0.8959

The rationale for this observation lies in the fact that, although China’s overall agricultural mechanisation has reached a high level, there are still regions, such as hills and mountains, that are not conducive to large-scale agricultural machinery production. In these areas, farmers engage in small-scale crop production, which does not yield significant income or subsidies. Consequently, in the absence of food subsidies buffering the reduction in income, even minor water pollution can lead to a notable decline in income and exacerbate the urban-rural income gap.

However, when water pollution reaches dangerously high levels, food security is jeopardized by the combined impact of low agricultural productivity and high pollution. This results in a sharp increase in food prices, ultimately narrowing the urban-rural income gap. Interestingly, once the level of agricultural productivity surpasses a certain threshold, the relationship between water pollution and the urban-rural income gap reverts to a "U" shaped pattern.

## Discussions and conclusion

### Discussions

This paper utilizes panel data from 30 provincial-level administrative regions in China, spanning the years 2010 to 2022, to consider water pollution as a factor of production and investigate its impact on the urban-rural income gap. After conducting validations, the research draws several conclusions. The findings reveal the following: (1) Water pollution emanating from non-agricultural sources exerts a U-shaped influence on the urban-rural income gap. Specifically, the deterioration of water quality that results in reduced crop production impacts the sales income of agricultural products, both in terms of quantity and price, ultimately leading to changes in the urban-rural income gap; (2) Mechanism analysis demonstrates that NASWP contribute to a decrease in per capita food production within a region, serving as a conduit for establishing a U-shaped relationship with the urban-rural income gap; (3) Threshold analysis further corroborates that water pollution arising from non-agricultural sources has a significant impact on the urban-rural income gap.

### Policy implications

This paper identifies a complex U-shaped relationship between water pollution and the urban-rural income gap, deepening our understanding of the Environmental Kuznets Curve theory and the theory of unbalanced growth. This relationship offers novel insights into addressing environmental pollution, narrowing the urban-rural income gap, and achieving shared prosperity. Drawing on these findings, we offer the following policy recommendations, aiming to provide valuable guidance for China in harmonizing environmental protection with urban-rural development.

Primarily, an emphasis on fortification of source management is of utmost importance, with an explicit focus on proficiently abating NASWP. In light of the ramifications of water pollution on the economic inequality between urban and rural locales, the onus of instituting effective and resourceful strategies to counteract this particular category of contamination cannot be overstated. An integral tactic in this regard involves the facilitation of technological progression and adoption of eco-friendly production protocol. This encompasses the stimulation of corporate entities to imbibe methodologies aligned with cleaner production, culminating in a significant decrease in pollutant egression. As part of a supplementary measure, the imposition of stringent water pollution effluent standards is necessitated, accompanied by an escalation in punitive repercussions for entities that do not comply, establishing a formidable regulatory structure. In culmination, it is essential to construct a comprehensive water quality surveillance system, programmed to monitor trends in water pollution expeditiously, allowing for the implementation of timely remedial actions to conserve water resources.

To ensure impactful policy development and execution, an enhancement in legislative backing and fortification of regulation is paramount. The government is tasked with ensuring the dissemination of policies that are not only relevant but also effective, whilst also bolstering the oversight and evaluation of the same. Principally, the formulation of bespoke policy measures, which are suitably tailored in accordance with the discrete conditions encountered in each region, is an essential step. By selecting pertinent policy support strategies that resonate with each region’s unique water contamination scenario and agricultural production features, the government can ascertain the relevance and potency of their enacted policies. Subsequently, it becomes vital to augment the supervisory and evaluative mechanisms pertaining to these policies. By implementing robust monitoring frameworks, any issues or deficiencies in policy enforcement can be promptly identified and addressed, thereby ensuring seamless and efficacious policy execution.

The paramountcy of advancing ecological development and unifying urban-rural dynamics, in the context of combatting water contamination, is resoundingly clear. Water pollution not only exacerbates economic disparities between metropolitan and rural areas but also jeopardizes citizens’ standard of living and the endurance of our ecological system. Therefore, fostering improved defensive and rehabilitative frameworks for our environment, while concurrently endorsing ecological progression and urban-rural communion, is of essential value. Initially, it is critical to allocate appropriate funds towards the minimization of water contamination, in addition to promoting initiatives such as reforestation and the recovery of aquatic bodies. Such endeavors will, in effect, shield our aquatic ecosystems, enhance the quality and availability of water, and ensure the safety of potable water and the general living conditions. Concomitantly, it is vital to amplify backing and direction for rural economies, advocating for the recalibration and refinement of the rural industrial framework. This would entail nurturing the growth of niche industries and rural tourism sectors, thereby diversifying the revenue streams for the farming population and encouraging the amalgamation of urban-rural economic growth. By accomplishing such an amalgamation, we pave the way for a lucrative and progressive scenario that is mutually beneficial for both our economy and the environment.

### Limitations and future research

This study is indeed constrained by several limitations. Firstly, our primary focus is on the impact of the current severity of water pollution on the urban-rural income gap. However, accurately defining the impact of the historical accumulation of pollutants discharged in wastewater on this income gap remains challenging. Secondly, our analysis cannot encompass all substances that contribute to the severity of water pollution, such as heavy metal substances. Thirdly, while this paper explains changes in the urban-rural income gap from the perspective of agricultural product consumption by urban and rural residents, it does not delve into other factors that may cause changes in this gap due to water pollution severity.

With the advancing practices in statistics and data mining, future research can utilize larger sample sizes within extended timeframes and develop methodologies that can appropriately account for pollutant stocks. Additionally, future studies can verify the relationship between the urban-rural income gap and the types of pollutants found in various wastewater sources, as well as their differing impacts on crops. Furthermore, it would be worthwhile to investigate the impact of water pollution on other non-agricultural avenues that affect the incomes of both rural and urban residents. By addressing these limitations, we can gain a more comprehensive understanding of the complex relationship between water pollution and the urban-rural income gap.

## Supporting information

S1 File Dataset used for regression analysis. (XLSX)
