# Correction: Examining the U-shaped relationship between non-agricultural sources water pollution and the urban-rural income gap

**DOI:** 10.1371/journal.pone.0320587

**Published:** 2025-03-10

**Authors:** Dong He, Zhongyuan Sheng, Chunxiao Tian

The authors’ current address is incorrect. The correct current address is: Xihua University, 9999 HongGuang Road, Pidu District.
